# To the Editors of the Medical and Physical Journal

**Published:** 1804-05-01

**Authors:** G. Wilkinson

**Affiliations:** Sunderland


### *To the Editors of the Medical and Physical Journal.*

Gentlemen,

Having received from several medical gentlemen, (particularly in London) specimens of barks enclosed in franks, of what was sold to them as the real *cortex salicis latifoliæ*,* and finding these to be no other than the common species used by basket-makers; I have deemed it highly necessary, to prevent imposition in future, to send to the different professors, lecturers, and teachers in London and Edinburgh,† specimens of the real bark, and the leaves of the tree, accompanied by the following circular letter.

"Sir,

"If you are disposed to notice to your pupils, the *cortex salicis latifolia*, I have sent you a specimen of the bark and leaves of the tree for that purpose. This is the more necessary, as the want of this information renders those who wish to try its effects liable to be imposed on by the vendors; who either may not know it, or, what is worse, sell other sorts in its stead, thereby bringing into disrepute a valuable indigenous substitute for the *cinchona*.

I am, &c.

G. WILKINSON."

*Sunderland, February* 13, 1804.

---

* The only genuine bark I can find in London, where I was in the month of June last, was at Gordon's herb shop, Newgate Market.

† Specimens have been sent to the Medical Schools of St. Bartholomew, St. Thomas, and the London Hospitals; to Drs. Pearson, Batty, Clark, Dennison, and Squires; Messrs. Brookes, Wilson, Thomas, Carlisle, &c. in London. To Drs. Monro, Gregory, Duncan, Hope, Home, Hamilton, Rutherford, and Mr. James Russel, Edinburgh.

A Case

